# Esculetin unveiled: Decoding its anti‐tumor potential through molecular mechanisms—A comprehensive review

**DOI:** 10.1002/cnr2.1948

**Published:** 2023-12-08

**Authors:** Md. Rezoan Hossain, Fatema Tuj Zahra Shova, Munni Akter, Shahporan Shuvo, Nasim Ahmed, Afroza Akter, Munira Haque, Umme Salma, Md Roman Mogal, Hasi Rani Saha, Bidhan Chandra Sarkar, Md Sohel

**Affiliations:** ^1^ Biochemistry and Molecular Biology Rajshahi University Rajshahi Bangladesh; ^2^ Biotechnology and Genetic Engineering Mawlana Bhashani Science and Technology University Tangail Bangladesh; ^3^ Department of Medical Dinajpure Nursing College (Affiliated Rajshahi University) Dinajpur Bangladesh; ^4^ Biochemistry and Molecular Biology Mawlana Bhashani Science and Technology University Tangail Bangladesh; ^5^ Department of Pharmacy Mawlana Bhashani Science and Technology University Tangail Bangladesh; ^6^ Departmnet of Microbiology Noakhali Science and Technology University Noakhali Bangladesh; ^7^ Biochemistry and Molecular Biology Primeasia University Dhaka Bangladesh

**Keywords:** anticancer mechanism, cancer types, esculetin, pharmacokinetic, synergistic

## Abstract

**Background:**

The growing complexity of cancer has made it a significant concern in the medical community. Although cancer research has advanced, it is still challenging to create new effective medications due to the limitations and side effects of existing treatment strategies. These are enforcing the development of some alternative drugs from natural compounds with fewer drawbacks and side effects.

**Aim:**

Therefore, this review aims to provide up‐to‐date, crucial, and all‐encompassing data on esculetin's anticancer activity, including all relevant molecular and cellular processes based on in vivo and in vitro investigations.

**Results:**

According to the literature review, esculetin is available in nature and is effective against 16 different types of cancer. The general mechanism shown by esculetin is modulating signaling cascades and its related pathways, like cell proliferation, cell growth, autophagy, apoptosis, necrosis, inflammation, angiogenesis, metastasis, invasion, and DNA damage. Nanoformulation of esculetin improves this natural product's efficacy by improving water solubility. Esculetin's synergistic effects with both natural substances and conventional treatments have been shown, and this method aids in reversing resistance mechanisms by modulating resistance‐related proteins. In addition, it has fewer side effects on humans than other phytochemicals and standard drugs with some good pharmacokinetic features.

**Conclusion:**

Therefore, until standard chemotherapeutics are available in pharmaceutical markets, esculetin should be used as a therapeutic drug against various cancer types.

## 1 INTRODUCTION

Cancer is a complex disease that affects millions of individuals worldwide each year.^1^ This is becoming a significant global health concern, and the number of people diagnosed and dying of cancer is growing at an alarming rate in both industrialized and developing nations.^2^, ^3^ Despite the advances in research, developing new effective medicines is still challenging due to limitations like—side effects like hematopoietic system toxicity, neurotoxicity, cardiotoxicity, nephrotoxicity, oral mucositis, and various gastrointestinal problems.^4^, ^5^, ^6^, ^7^, ^8^, ^9^ This has led researchers to explore the potential of natural products as anticancer agents.^10^, ^11^, ^12^, ^13^ Various medicinal plants have been used as alternative therapies for cancer patients. Because of their chemical variety, natural products have been studied for over 50 years for their ability to fight cancer. This means that using recently identified chemicals derived from plants may offer an innovative and reliable therapeutic ingredient for healing a broad range of human cancers.^14^

One such natural compound that has recently gained attention for its potential anticancer properties is esculetin. Esculetin is a phytoestrogen found in a wide variety of herbs and plants. Several studies have investigated the possible anticancer activity of esculetin in various human malignancies such as breast,^15^ colon,^16^ liver,^17^ pancreatic,^18^ leukemia,^19^ lung,^20^ laryngeal,^21^ oral,^22^ salivary,^23^ melanoma,^24^ prostate,^25^ renal,^26^ endometrial,^27^ gastric,^28^ osteosarcoma,^29^ ovarian cancer.^30^ The mechanism of action of esculetin in inhibiting cancer cell growth has been linked to its ability to interfere with enormous signaling pathways that regulate cell division, differentiation, and survival.^21^ Esculetin's interaction with a wide variety of regulatory molecules and regulators mediates its broad spectrum of anticancer effects.^18^ These results imply that esculetin might be effective in the treatment and prevention of cancer and in the creation of novel medications.

Although esculetin has been the subject of several in vitro, in vivo, and pre‐clinical trials, including a wide variety of human malignancies, a comprehensive review with mechanistic insight has not been reviewed yet. Therefore, we aimed to highlight the overall anticancer effects of esculetin against numerous human malignancies, including its relation with structure and anticancer perspectives in different study models. Moreover, we targeted unraveling synergistic mechanisms with other natural products and traditional drugs currently used, overcoming resistance patterns and existing drug resistance activities, and developing nano‐strategies for better efficacies, with some additional information, including a pharmacokinetics study and toxicity profile. Therefore, the information that will be covered here will encourage potent scholars to design novel and potential drugs to fight against cancerous neoplasm.

## 2 SOURCES OF ESCULETIN

Esculetin is available in several plants, including *Aesculus hippocastanum* L, *Ceratostigma willmottianum*, and *Citrus limonia*.^31^ The intramolecular cyclization of a cinnamic acid derivative yields this naturally occurring lactone. It may be found as caffeic acid conjugates and glycosides in chicory as well as many other poisonous and therapeutic plants.^32^ It is also found in *Artemisia pereiopods, Euphorbia decipiens*,^33^ *Nicotiana tabacum*,^34^ *Fraxinus rhynchophylla hance, Osbeck rutaceae, Euphorbia lathyris* L, *Ceratostigma willmottianum, Artemisia capillaris, Viola yedoensis makino*.^35^ Esculetin can be biologically synthesized from *Escherichia coli*. *Escherichia coli* expressing the genes F6′H (encoding feruloyl CoA 6′ hydroxylase) and 4CL (encoding 4‐coumarate CoA: ligase) were grown in media containing p‐coumaric acid, caffeic acid, and ferulic acid, resulting in the synthesis of the coumarins umbelliferone, esculetin, and scopoletin. The summary of esculetin source is shortlisted in Table 1.

**TABLE 1:** Reported plant sources of Esculetin.

Name of plants species	References
*Aesculus hippocastanum* L.	^31^
*Ceratostigma willmottianum*	^31^
*Citrus limonia*	^31^
*Artemisia eriopoda*	^33^
*Euphorbia decipiens*	^33^
*Nicotiana tabacum*	^34^
*Fraxinus rhynchophylla hance*	^35^
*Osbeck rutaceae*	^35^
*Euphorbia lathyris* L	^35^
*Ceratostigma willmottianum*	^35^
*Artemisia capillaris*	^35^
*Viola yedoensis makino*	^35^

## 3 CHEMISTRY OF ESCULETIN

**FIGURE 1:**
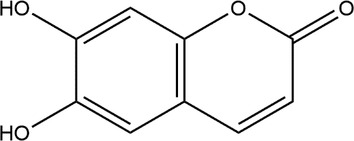
Chemical structure of esculetin.

Esculetin is known as 6,7‐dihydroxychromen‐2‐one (IUPAC nomenclature) and has the molecular formula C_9_H_6_O_4._ Esculetin is a hydroxycoumarin structurally similar to umbelliferone but has a hydroxy group replacing the hydrogen at position 6. Its metabolite is found in plants and has important roles as an antioxidant and UV filter. There are 13 heavy atoms, 1 covalently bound unit, 66.8 Å^2^ of topological polar surface area, zero net formal charges, and zero isotope atom counting. This substance has a very heavy molecular weight of 178.14 g/mol. However, the precise value for the mass per molecular unit is 178.0266 g. The chemical structure of esculetin is drawn in Figure 1.

## 4 ANTICANCER ACTIVITY OF ESCULETIN ON VARIOUS TYPES

**FIGURE 2:**
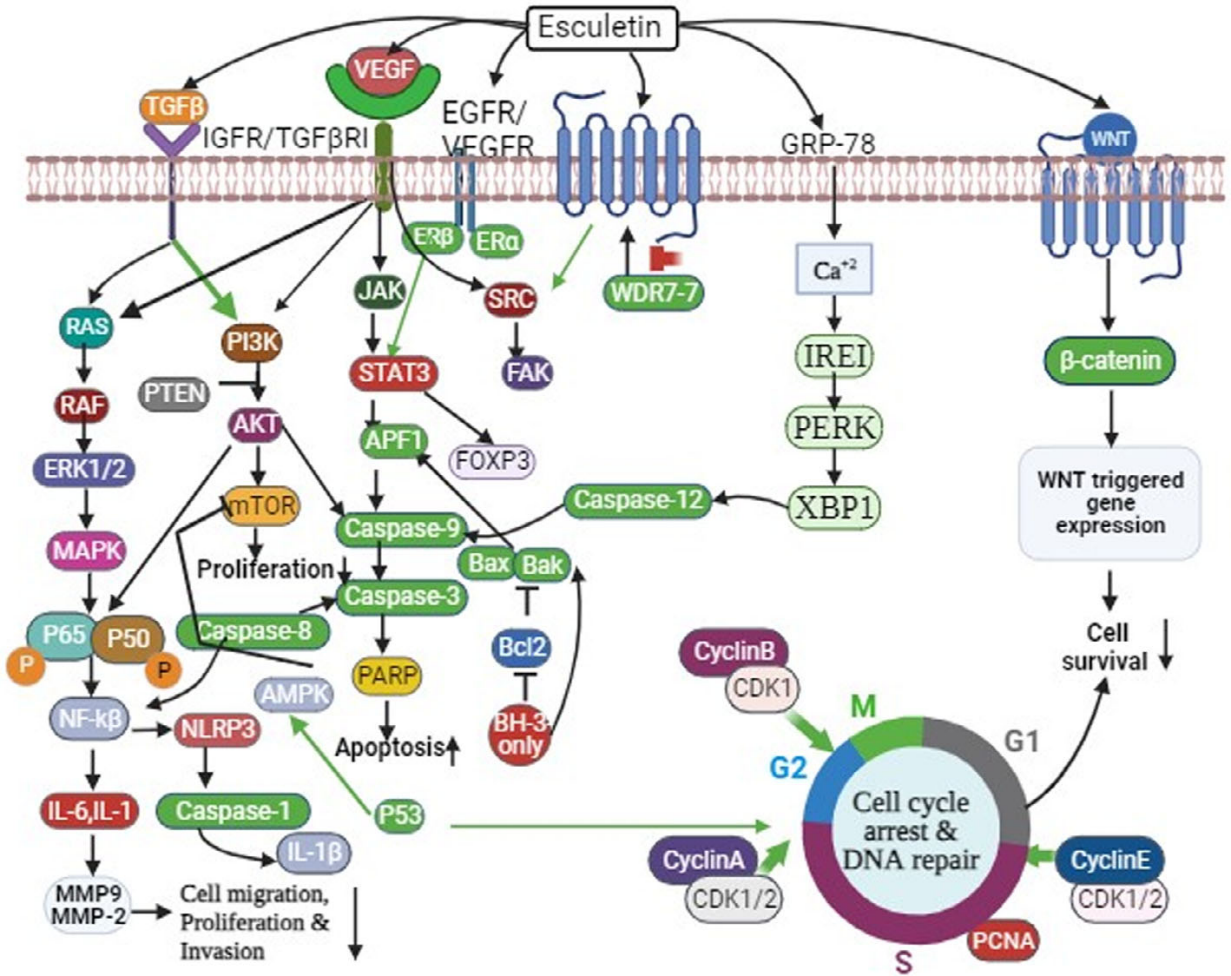
The molecular cascade of esculetin regarding anti‐cancer mechanism against human malignancies. These pathways are interconnected; activating one pathway either accelerates to other or inhibits another mechanism. The ultimate goal is to control carcinogenic pathways.

Various experimental studies exhibited that esculetin is effective against human malignancies by regulating various mechanisms, including cell proliferation, cell growth, autophagy, apoptosis, necrosis, inflammation, angiogenesis, metastasis, invasion, and DNA damage (Figure 2). A summary of the chemotherapeutic activity of esculetin against human malignancies is presented in Table 2.

**TABLE 2:** Summary of chemotherapeutic activity of Esculetin against numerous cancer.

Cancer type	Dose	Type of study (in vitro and in vivo)	Molecular mechanism	Molecular target	References
Breast cancer	1 mg/mL 1–10 mg/kg	In vitro Caco‐2 In vivo FVB mice	N/A	↑BCRP ↑MRP2	^36^
	0–60 μM	In vitro MCF‐7, MDA‐MB‐231, ZR‐75‐1	↑Cytotoxicity ↑Apoptosis	↑G2/M cell cycle arrest ↓MMP and cytochrome C ↑Caspase‐9/caspase‐3 ↑p21, p53 expression ↓CDK1 expression ↓Cyclin B1 ↑Intracellular Ca^2+^ concentration	^15^
	40–80 ng/mL	In vitro MDA‐MB‐231	↓Cell growth ↓Cell proliferation	↓Secretion of linoleic acid	^37^
	0–1 mM	In vitro CMT‐U27, CF41	↑Apoptosis ↓Migration ↓Cell viability	↑G0/G1 and S phase cell cycle arrest ↓Cyclin D1 expression ↓CDK4 expression ↑Caspase‐3	^38^
Colon cancer	0–600 μM	In vitro LoVo	↓Cell proliferation ↑Apoptosis	↑G0/G1 phase cell cycle arrest ↑p53, p27, p21 expression ↑Bax expression ↑Caspase‐9,3,7 ↓Bcl‐2 expression	^16^
	0–2000 μg/mL	In vitro HT‐29, SW480, HCT‐116, Caco‐2	↓Migration ↓Cell proliferation	↓Ki67, N‐cadherin, E‐cadherin, β‐catenin, c‐Myc ↓Cyclin D1, MMP2, MMP7 ↓VEGF, Wnt3	^40^
		In vivo Male Fischer rat	↓Oxidative DNA damage ↓Carcinogenesis	N/A	^41^
	55 μg/mL	In vitro HT‐29	↓Cell viability ↑Apoptosis	↓Bcl‐2 expression ↑Caspase‐9/caspase‐3 ↑Cyt C release ↑ROS formation and MAPK ↑Mitochondrial membrane depolarization	^42^
	55 μg/mL	In vitro HT‐29, HCT‐116	↑Apoptosis	↑GRP‐78 ↑IRE1 ↑PERK ↑XBP1 ↑Caspase‐12 ↑Mitochondrial Ca^2+^ level	^43^
	10–30 mg/kg	In vitro HCT‐116, SW480, LS174T, HCT15 In vivo Male nude mice	↓Invasion ↓Cell proliferation ↓Migration ↓Cell growth	↓Wnt/β‐catenin ↓Axin2	^44^
	0–24, 0–300 μg/mL	In vitro HCT‐116	↓Cell growth ↓Cell proliferation	↑G1 phase cell cycle arrest ↑p27KIP ↑ERK1/2 ↓CDK4 ↓Cyclin D1	^45^
	20–100 mg/kg	In vitro HCT‐116 In vivo Female athymic nude mice	↓Cell growth ↓Cell viability	↓Wnt/β‐catenin ↓β‐catenin/Tcf complex	^46^
	500 mg/kg	In vivo Male Wistar rat	↓Angiogenesis ↓Cell proliferation	↓c‐Myc	^47^
	0.56–2.24 mmol/L	In vitro SW480	*↓Proliferation* ↓Tumor growth	↓Wnt signaling pathway. ↓β‐catenin, c‐Myc and cyclin D1	^48^
Liver cancer	0.01–100 g	In vivo Male C57BL/6N mice	↓Hypertrophy ↓Lipid accumulation ↓Inflammation	↓Fasn, Dgat2, Pap ↓Tlr4, Myd88, TNF‐α, IL‐6 ↓MCP‐1	^50^
	6 mg/kg	In vivo Male Swiss and Wistar mice	↑Anti‐hepatotoxicity ↓Lethality ↓Serum enzyme	N/A	^51^
	10–100 μM	In vitro HepG2 In vivo Male C57BL/6J mice	↓Serum enzyme ↓Serum lipid	↑Nrf2 ↓GSH, MDA	^52^
	0–300 μg/mL	In vitro SMMC‐7721	↑Apoptosis ↓Cell proliferation	↑Caspase 9, 3 ↑Bax ↓Bcl‐2 ↓Akt, PI3K, IGF‐3	^17^
	0.5–5 mg/kg	In vivo Male Sprague–Dawley rat	↑Leukocyte infiltration ↑Necrosis	↓MDA ↓LDH ↓ALT	^53^
		In vivo Male Wistar rat	↓Serum enzyme (ALT) ↓Lipid peroxidation	↑NQO1 ↑GSTP1	^54^
	0–4.48 mM 200–700 mg/kg	In vitro SMMC‐7721 In vivo C57BL/6J mice	↑Apoptosis *↓Proliferation* ↓Tumor growth	↑S phase cell cycle arrest ↑Caspase‐3 and caspase‐9 ↑Bax expression ↓Bcl‐2 expression	^55^
	50–500 μmol/L	In vitro SMMC‐7721	*↓Proliferation* ↓Cell viability	↓Wnt/β‐catenin signaling pathway ↓β‐catenin, c‐Myc and cyclin D1	^56^
Pancreatic cancer	100 μM	In vitro PANC‐1, MIA PaCA‐2, AsPC‐1	↓Cell growth ↑Apoptosis	↑G1‐phase cell cycle arrest ↑Caspase 3, 8, 9 ↓ROS ↓p65‐NF‐κB	^18^
Leukemia	20 μM	In vitro HL‐60	↓Proliferation ↑Autophagy ↑Apoptosis ↓Cell growth	↑G0/G1 cell cycle arrest ↓Cyclin D1 and D3 ↓Raf/MEK/ERK signaling pathway	^60^
	30 μg/mL	In vitro U937	↑Apoptosis	↑DR4 ↑Bid cleavage ↓MMP	^61^
	100‐500 μM	In vitro NB4	↑Apoptosis	↓NFκB p65 ↑NFκB p50 ↑Nrf2	^62^
	30 μg/mL	In vitro U937	↓Cell viability ↓Cell growth	↑G1 cell cycle arrest ↓pRB phosphorylation ↓p21, cyclin E	^63^
	50‐100 μM	In vitro U937	↓Proliferation ↑Apoptosis	↓MAPKs activation ↑Bid cleavage, Bax, cytochrome C ↑MEK/ERK and JNK pathways ↓Intracellular GSH levels	^19^
	20–500 μM	In vitro NB4	↓Cell viability ↑Apoptosis ↑DNA fragmentation	↑Caspase‐3, ‐9 ↓Bcl‐2/Bax ratio ↑Phosphorylated‐Akt ↑Superoxide anion ↓GSH level	^64^
	30 μg/mL	In vitro U937	↓Cell viability ↑Apoptosis	↓Bcl‐2 ↑Cytochrome C ↑ERK and JNK	^65^
	100 μM	In vitro Kasumi‐1	↓Proliferation	↓AML1‐ETO expression ↓C‐Kit mRNA ↑LAT1 and RUNX3	^66^
Lung cancer	0–100 μg/mL	In vitro NCI‐H358 and NCI‐H1299	↓Proliferation ↑Apoptotic effect	↑p27, p21 ↓Sp1 ↓Survivin	^20^
	0–80 μmol/L, 100 mg/kg	In vivo LLC	↓Proliferation	↓c‐Myc ↓Cyclin D1 and NF‐κB ↓Wnt Targeted Genes	^69^
	5,20 μM	In vitro A549	↓Proliferation	↓Vimentin and Snail mRNAs ↑E‐cadherin expression	^70^
Laryngeal Cancer	0‐0 μM 50–100 mg/kg	In vitro and in vivo Hep‐2	↓Proliferation, migration, and invasion	↑G1/S cell cycle arrest ↓Phosphorylation of STAT3	^21^
Oral cancer	5–20 μg/mL	In vitro HN22 and HSC4	↓Cell growth ↓Cell viability ↑Apoptosis	↓Sp1 ↑G1 cell cycle arrest	^22^
	10 μg/mL	In vitro SAS	↑Apoptosis	↑G2/M cell cycle arrest ↑DR5, caspase‐ 8	^79^
	0–20 mg/mL	In vitro HN22 and HSC2	↑Apoptosis ↓Proliferation ↓Cell viability	↓EGFR/PI3K/Akt signaling pathway ↓Nucleophosmin (NPM)	^80^
Salivary cancer	50–150 μM 100 mg/kg	In vitro and in vivo A253	↓Proliferation	↑Bax, caspase‐ 3, ‐9 ↑Poly (ADP‐ribose) polymerase ↓Bcl‐2	^23^
Melanoma	0–80 μg/mL	In vitro G361	↑Nuclear shrinkage and fragmentation ↑Apoptosis	↓Sp1 ↑p27, p21, bax, caspase‐3 ↓Cyclin D1, procaspase‐3 and PARP	^24^
Prostate cancer	0–600 μM	In vitro PC3, DU145, and LNCaP	↓Cell migration ↓Cell survival ↑Apoptosis ↓Proliferation	↑G1 cell cycle arrest ↑Cytochrome C, p53, p21, p27 ↓Cdk2, Cdk4 ↓Akt phosphorylation	^25^
Renal cancer	0–200 μg/mL	In vitro 786‐O and SN12‐PM6	↓Cell migration and invasion ↑Apoptosis	↑G0/G1 and G2/M cell cycle arrest ↓Cyclin D1, CDK4, CDK6, and c‐Myc expression ↑E‐Cadherin, caspase‐3 ↓N‐cadherin and vimentin	^26^
Endometrial cancer	0–120 μM, 100 mg/kg	In vitro and in vivo HEC‐1B and Ishikawa	↓Proliferation ↑Apoptosis	↓BCLXL, XIAP, and pAKT ↑Caspase‐3 and PARP	^27^
Gastric cancer	30–80 μM	In vitro Human cell line GES‐1, SGC‐7901, MGC‐803, BGC‐823.	↑Apoptosis ↑Cytotoxicity ↓Cell growth ↓Cell viability	↑caspase‐3 ↑caspase‐8 ↑caspase‐9 ↑Bax and Bak ↓Bcl‐2 and Bcl‐xL **↑**CypD **↑**ROS ↓MMP	^28^
	850 μM	In vitro Human cell line MGC‐803, HGC‐27 and BGC‐823, GES‐1.	↑Apoptosis ↓Cell proliferation	↑caspase‐3 ↑caspase‐9 ↑Bax/Bcl‐2 ratio ↑Cyt‐C release ↓IGF‐1, p‐PI3K, p‐Akt ↓MMP	^74^
	50–100 mg/kg	In vivo Female BALB/c mice.	↓Cell growth ↑Apoptosis **↓**Tumor weight **↓**tumor size ↓Cell proliferation	↑Caspase‐3 ↓Bcl‐2 ↓Nucleus Ki‐67 ↓IGF‐1, p‐PI3K, p‐Akt	^74^
Ovarian Cancer	N/A	In vitro HO‐8910	↑Apoptosis ↓Proliferation	↑Caspases 3,9 ↑Bax/Bcl‐2	^82^
	N/A	In Vitro	↑Apoptosis ↓Cell viability, migration, invasion	↓JAK2/STAT3 ↑ROS ↑G0\G1 cell cycle arrest	^83^
Osteosarcoma	20–100 μM	In vitro and in vivo LM8	↓Cell proliferation ↓Tumor growth ↓Metastasis	↑G1 cell cycle arrest ↓Cyclin D1, CDK4 and MMP‐2 ↓TGF‐β1 and VEGF ↓IL‐10, MCP‐1, STAT3	^29^

### 4.1 Breast cancer

Breast cancer is the most common kind of cancer in women. Many investigations uncovered that esculetin has chemotherapeutic activity against this type of cancer. Breast cancer resistance protein (BCRP) and multidrug resistance‐associated protein 2 (MRP2) were affected by esculetin‐7‐O‐Glucoronide and 4‐Methylesculetin‐7‐O‐Glucoronide (1 mg/mL −10 mg/kg), as determined by in vitro and in vivo studies using Caco‐2 cell line and FVB mice, respectively.^36^ Esculetin (0–60 μM) administration was found to induce apoptosis and cytotoxicity via upregulation of p21, p53, caspase‐3, ‐9, and cytC and downregulation of CDK1 and cyclin B1 expression in a separate in vitro study using the MCF‐7, ZR‐75‐1, and MDA‐MB‐231 cell lines.^15^ Esculetin inhibited the growth of cancer cells, linoleic acid production, and cell proliferation when administered to MDA‐MB‐231 cancer cells at dosages of 40, 80, and 90 ng/mL.^37^ According to Choi et al CMT‐U27, CF41.mg canine mammary gland tumor cells undergo apoptosis, and G0/G1 and S phase cell cycle arrest when exposed to esculetin at concentrations between 0 and 1 mM, and the expression of the cell death protein caspase‐3 is upregulated by esculetin while the expression of the cell cycle regulators CDK4 and cyclin D1 is downregulated.^38^

In conclusion, it is clear from the current mechanistic investigations of breast cancer that esculetin promotes the overexpression of tumor suppressor genes that can control cell cycle progression. This naturally occurring coumarin chemical also regulates the expression of numerous genes. Esculetin is also capable of inhibiting the growth and spread of malignant cells.

### 4.2 Colon cancer

Colon cancer is the third most frequent cancer overall and the second most significant cause of cancer mortality.^39^ Esculetin inhibits proliferation in an in vitro investigation of LoVo cells by inducing apoptosis and causing a G0/G1 cell cycle arrest characterized by increased expression of Bax, p53, p27, p21, and caspase‐9,3,7 and decreased expression of Bcl‐2.^16^ Researchers found that esculetin inhibited the migration and proliferation of HT‐29, SW480, HCT‐116, and Caco‐2 cell lines by downregulating Ki67, N‐cadherin, E‐cadherin, β‐catenin, c‐Myc, cyclin D1, MMP2, MMP7, VEGF, and Wnt3a at concentrations of 0.2, 2, 20, 200, and 2000 μg/mL.^40^ Kaneko et al revealed that esculetin (0.01%, 0.02%, 0.05%) treatment in male Fischer rats prevents oxidative damage and carcinogenesis.^41^ According to another in vitro study, esculetin at 55 μg/mL plays a critical role in inhibiting cancer cell viability and inducing apoptosis by increasing Caspase‐3 and ‐9 activation and the level of reactive oxygen species (ROS) and cyt‐C release while decreasing antiapoptotic protein Bcl‐2 expression.^42^ Esculetin strongly caused apoptosis and increased mitochondrial Ca^2+^ levels through elevation of GRP‐78, IRE1, PERK, and XBP1, and pro‐apoptotic factor caspase‐12, according to a study by Kim et al on HT‐29 and HCT‐116 cells at 55 μg/mL.^43^ Using HCT‐116, SW480, LS174T, HCT15, and male nude mice, researchers found that esculetin (10, 30 mg/kg of body weight) decreased the invasion and metastasis, growth, and proliferation of colon cancer cells by downregulating Wnt and Axin2 and reduction of β‐catenin.^44^ By upregulating p27KIP and ERK1/2 and downregulating cyclin D1 and CDK4, esculetin's anti‐proliferative actions on HCT‐116 cells caused a G1 cell cycle arrest and decreased cell proliferation and growth.^45^ Lee et al experimented both in vitro on HCT‐116 cells and in vivo female athymic nude mice and stated that by inhibiting Wnt/β‐catenin and β‐catenin/Tcf complex, esculetin reduces the number and survival of colon cancer cells.^46^ Esculetin (500 mg/kg of body weight) reduced cell proliferation and angiogenesis in male Wistar rats while downregulating c‐Myc.^47^ The downregulation of c‐Myc, cyclin D1, and β‐catenin by esculetin in the SW480 cell line in vitro results in decreased cell proliferation and tumor growth.^48^

In summary, esculetin's ability to kill cells via mediating apoptosis‐related cell death and regulating multiple signaling pathways suggests that it may be helpful as a treatment against colon malignancy.

### 4.3 Liver cancer

Of all the known malignant tumors, liver cancer is notable for its wide variety of manifestations. Chronic liver fibrosis and inflammation are major risk factors for the development of hepatocellular carcinoma (HCC), which may arise in a number of ways from patient to patient.^49^ Results from an in vivo investigation conducted by Choi et al on male C57BL/6N mice showed that esculetin (0.01/100 g), reduces hypertrophy, lipid accumulation, and inflammation by downregulating Fasn, Dgat2, Pap, Tlr4, Myd88, and lowering TNF‐α, Il6, and MCP‐1.^50^ Similarly, another in vivo study with male Swiss and Wistar mice proved that esculetin (6 mg/kg) reduced lethality, increased anti‐hepatotoxicity, and prevented serum enzyme ALP, AST, and ALT levels.^51^ In vitro study on HepG2 cells and in vivo study on male C57BL/6J mice with esculetin (10, 50, 100 μM, and 200 mg/kg, respectively) showed that esculetin decreased the level of ALP, AST, and ALT as well as serum lipids through increasing Nrf2 signaling^52^ Juan Li et al performed research on SMMC‐7721 cells to estimate the effect of esculetin at 0, 25, 50, 100, 200, and 300 μg/mL concentrations and found that esculetin elevated caspase 9, 3, and Bax and downregulated Bcl‐2, Akt, PI3K, and IGF‐3 to cause apoptosis and limit cell proliferation.^17^ Esculetin inhibited the release of lactate dehydrogenase (LDH) and alanine transaminase (ALT) at concentrations of 5–20 μg/mL, as well as the generation of malondialdehyde (MDA) and alanine aminotransferase (AST) at concentrations of 0.5, 5 mg per kg, and decreased oxidative stress and increased necrosis in male Sprague‐Daw rat.^53^ Another in vivo study on male Wistar rats demonstrated that esculetin (0.5% wt/wt) decreased ALT, hepatic lipid peroxidation, and liver damage while increasing NQO1 and GSTP.^54^ Esculetin has been shown to cause apoptosis and decrease proliferation and tumor development in both in vitro and in vivo experiments by causing a cell cycle arrest in the S phase, where Bax, caspase‐3, and ‐9 are elevated, and Bcl‐2 is downregulated.^55^ The downregulation of c‐Myc, cyclin D1, and β‐catenin by esculetin in SMMC‐7721 cells in vitro results in decreased cell viability and proliferation.^56^

To sum up, activating the death receptor and its downstream pathways and managing oxidative stress by antioxidant‐related enzymes allow esculetin to prevent the proliferation of liver cancer cells. Moreover, this substance controls growth factors and receptors linked to cancer, reducing some signaling pathways and inflammation.

### 4.4 Leukemia

According to GLOBOCAN, leukemia rated fifteenth, and eleventh in terms of cancer incidence and mortality in 2018. On a global scale, men are more affected by leukemia than females. GBD predicts that the worldwide incidence of leukemia rose by 26% between 2005 and 2015.^57^ While leukemia accounts for about 3% of all malignancies, it continues to be one of the leading causes of fatalities related to cancer in adolescents, and those under the age of 40.^58^ Esculetin is a significant coumarin with significant anticancer potential.^59^ 20 μM dosage of esculetin promoted apoptosis, and decreased cell proliferation and growth in an in vitro research employing HL‐60 cells by lowering Cyclin D1 and D3 expression, causing a cell cycle arrest in the G0/G1 phase, and inhibiting the Raf/MEK/ERK signaling cascade.^60^ Another investigation on the U937 cell line by Park et al showed that esculetin promotes apoptosis by activating caspase, upregulating Bid cleavage, and death receptor 4 (DR4) expression when delivered at 30 μg/mL.^61^ When esculetin was administered at a concentration of 100 μM for 19 h, NF‐κB p65 was reduced, nuclear levels of NF‐κB p50 and Nrf2 were raised, and apoptosis was triggered.^62^ Another in vitro investigation on U937 cells showed that esculetin (30 μg/mL) reduces the proliferation and survival of cells by downregulating p21 and cyclin E expression and promoting G1 cell cycle arrest.^63^ Lin et al investigated the impact of esculetin (50–100 μM) on U937 cells for 24, 48, and 72 h and discovered that esculetin promoted apoptosis and suppressed cell growth by lowering intracellular GSH levels, enhanced Bax, Bid cleavage, and release of cytochrome C, and triggered the MEK/ERK and JNK signaling pathways.^19^ Esculetin decreases cell viability and proliferation and enhances apoptosis and DNA fragmentation by activating caspase‐3 and ‐9, with time‐dependent upregulation of phosphorylated‐Akt and superoxide anion and downregulation of Bcl‐2/Bax ratio and Glutathione (GSH) level.^64^ Park et al concluded that esculetin inhibited the proliferation of human leukemia U937 cells via mitochondrial‐mediated apoptosis induction by increasing Cytochrome c release and phosphorylation of extracellular‐regulated kinase (ERK) and c‐Jun *N‐terminal kinase* (JNK) while simultaneously downregulating the anti‐apoptotic protein Bcl‐2.^65^ Esculetin reduces proliferation in the Kasumi‐1 cell line by suppressing C‐Kit mRNA and AML1‐ETO expression.^66^ A recent study conducted by Mathur A et al reported that, esculetin act as an anticancer agent by removing maturation arrest and downregulating canonical Wnt axis and upregulating of non‐canonical axis associated genes in leukemic blast cells.^67^

### 4.5 Lung cancer

Lung cancer is the most lethal kind of cancer among both men, and women in the United States. Additionally, it is the leading cause of death from cancer in men and the second leading cause of death from cancer in women globally.^68^ Human NCI‐H358 and NCI‐H1299 cell lines shown in vitro that esculetin (0–100) μg/mL promotes apoptosis, and suppresses cell proliferation by upregulation of p27 and p21 and downregulation of survival and Sp1.^20^ Zhu et al conducted study on Murine Lewis lung cancer (LLC) cells at a dosage of 100 mg/kg esculetin for 20 days and determined that esculetin inhibited cell proliferation by decreasing the expression of NF‐κB, c‐Myc, and Cyclin D1.^69^ Esculetin suppresses proliferation by downregulating Snail mRNAs and Vimentin expression and upregulating E‐cadherin expression in a dose‐dependent manner.^70^

### 4.6 Gastric cancer

Gastric cancer is one of the deadliest and most complicated illnesses, also known as stomach cancer. It has two primary locations, proximal and distal, and is more common in males.^71^, ^72^ Pre‐malignant stomach lesions, socioeconomic status, environment, food and lifestyle, genetics, *Helicobacter pylori*, Epstein–Barr virus, and smoking are some of the factors that contribute to the development of gastric cancer.^73^ According to several studies, it was found that esculetin is a natural stomach cancer therapeutic agent. Wanga et al showed both in vitro and in vivo that esculetin inhibits cell proliferation and increases apoptosis by way of the IGF‐1/PI3K/Akt signaling pathway, with upregulation of caspase‐3, and caspase‐9 expression as well as the Bax/Bcl‐2 ratio and cytC expression, and downregulation of IGF‐1, p‐PI3K, and p‐Akt expression.^74^ Pan et al also found that esculetin, when applied to human gastric cancer cells in vitro (30–80 μM), increases caspase‐3, caspase‐8, caspase‐9, Bax and Bak, CypD, ROS, and decreases expression of Bcl‐2 and Bcl‐xL, resulting in apoptosis and a reduction in cell growth and viability.^28^

Since, esculetin inhibits cancer cell migration and invasion by decreasing growth factor expression, and increasing expression of tumor suppressor proteins; it can be a promising therapeutic agent for gastric cancer.

### 4.7 Pancreatic cancer

Pancreatic cancer is fatal because it is often diagnosed at a late stage when treatment is no longer effective.^75^ In an in vitro investigation, PANC‐1, AsPC‐1, and MIA PaCA‐2 cells were shown to be sensitive to esculetin (100 μM), which triggered apoptosis and inhibited cell growth by activating caspases 9, 8, and 3, lowering p65‐ NF‐κB protein and ROS levels, and inducing a G1‐phase cell cycle arrest.^18^

We can say that esculetin is a promising prospective treatment agent for pancreatic cancer.

### 4.8 Laryngeal cancer

Laryngeal carcinoma is among the most common cancers of the respiratory system. Laryngeal cancer is one of the rare oncologic disorders whose 5‐year survival rate has declined over the previous four decades.^76^ Researchers Zhang et al found that esculetin (50–100 mg/kg) triggered G1/S phase cell cycle arrest and reduced STAT3 phosphorylation, leading to decreased proliferation, migration, and invasion at in vitro experiments using a Hep‐2 cell line and an in vivo nude mouse xenograft animal model based on the JAK‐STAT signaling pathway.^21^

Because esculetin displays anticancer characteristics through various pathways, it can potentially be used as a treatment for laryngeal cancer.

### 4.9 Oral cancer

Among all cancers, oral cancer holds the sixth highest incidence rate.^77^ Oral cancer is a worldwide concern with an incidence of 300 000, with around 55% of patients dying due to its advanced state upon presentation.^78^ Esculetin (6,7‐dihydroxy coumarin) is a coumarin molecule that suppresses cell proliferation, triggers apoptosis in various kinds of human cancer cells, and is a potential chemotherapeutic treatment. Cho et al investigated oral squamous cell carcinoma (OSCC) cell lines HN22 and HSC4 at concentrations of 5–20 μg/mL over 24–48 h and found that esculetin decreased cell growth, cell survival and promoted apoptosis through inhibiting Sp1 and induced G1 phase cell cycle arrest.^22^ At 10 μg/mL, esculetin increased caspase‐8 and DR5 protein expression, triggering apoptosis in the SAS cell line.^79^ Reducing Nucleophosmin (NPM) expression in HN22, and HSC2 oral squamous cell lines showed that esculetin decreased cell proliferation and induced death through the EGFR/PI3K/Akt signaling pathway.^80^

As a whole, esculetin has the potential to act as an anticancer property by targeting intracellular molecules to promote apoptosis and inhibit the proliferation and growth of oral cancer cells.

### 4.10 Ovarian cancer

Ovarian cancer is the worst and most deadly form of gynecologic cancer.^81^ However, like other cancers, esculetin has an anticancer effect on ovarian cancer. Esculetin induced apoptosis and inhibited cell proliferation in HO‐8910 cells in vitro by enhancing the expression of caspase‐3, ‐9, and Bax/Bcl‐2.^82^ Esculetin had shown to diminish cell viability and promote apoptosis via a G0/G1 cell cycle arrest characterized by increased ROS generation in both in vitro and in vivo studies.^83^

In conclusion, esculetin has the potential to function as a therapeutic agent for the treatment of ovarian cancer by inhibiting cell growth and triggering apoptosis via the activation of caspases and the JAK2/STAT3 signaling pathway.

### 4.11 Salivary cancer

Cancers of the salivary glands are very rare, making up just around 5% of all malignancies in the head and neck.^84^ Esculetin inhibited the proliferation of human submandibular salivary gland cancer cells by reducing Bcl‐2 expression and increasing Bax expression, as well as cleaved caspase‐3, caspase‐9, and Poly (ADP‐ribose) polymerase, according to an in vivo and in vitro study conducted by Park and colleagues using the A253 cell line.^23^

Finally, the results from both in vivo and in vitro experiments suggest that esculetin has pharmacological effects against salivary gland cancer by reducing the expression of Bcl‐2, a protein linked to the induction of apoptosis in tumor cells.

### 4.12 Melanoma

The most lethal form of skin cancer is melanomas. It may develop from a number of different factors, the most common of which is prolonged exposure to ultraviolet (UV) radiation from the sun, tanning beds, or tanning lamps.^85^ Human malignant melanoma G361 cells were studied in vitro with esculetin, and the results showed that esculetin (0–80 μg/mL) causes apoptosis and nuclear shrinkage and fragmentation in G361 cells by raising levels of p21, p27, Bax, and active caspase 3, while lowering levels of cyclinD1, procaspase 3, and PARP.^24^

In short, esculetin seems to block Sp1's ability to promote cell growth and cause apoptosis through Sp1 target proteins.

### 4.13 Prostate cancer

Prostate cancer is the second most common cancer in men globally, with a higher incidence in older age groups; risk factors include age, family history, and race.^86^ Turkekul et al reported that esculetin (0–600 μM) was associated with cell cycle arrest in the G1 phase, elevated cytochrome C, p53, p21, and p27 expression, and decreased Cdk2, Cdk4, and Akt phosphorylation, which induced apoptosis and inhibited cell migration, cell survival, and cell proliferation.^25^

In summary, the findings of this investigation provide insight into the therapeutic potential of esculetin. By triggering apoptosis and encouraging cell cycle arrest, esculetin significantly suppresses cell growth.

### 4.14 Renal cancer

Renal cell carcinoma (RCC) is the third most prevalent genitourinary cancer.^87^ Esculetin reduces renal cancer cell growth, migration, and invasion. Experiments using the 786‐O and SN12‐PM6 cell lines for RCC showed that esculetin‐induced apoptosis and cell cycle arrest at the G0/G1, and G2/M phases at concentrations of 0100200 μg/mL by increasing the expression of activated caspase‐3 and E‐Cadherin, and decreasing the expression of cyclin D1, CDK4, CDK6, c‐Myc, N‐cadherin, and vimentin.^26^

In brief, esculetin found in nature has anticancer capabilities due to its ability to modulate apoptosis‐related proteins.

### 4.15 Osteosarcoma

Osteosarcoma is an uncommon form of bone cancer that occurs in 3.4 persons per million annually around the globe.^88^ In this investigation, esculetin (20–100 μM) reduced LM8 cell proliferation and metastasis by blocking the expression of Cyclin D1, CDK‐4, and MMP‐2 and generating TGF‐1 and VEGF.^29^

### 4.16 Endometrial cancer

Endometrial cancer is prevalent, among the second most frequent gynecological cancers worldwide.^89^ A mice Xenograft model and in vitro studies on HEC‐1B cells showed that esculetin (0–120 μM) inhibited the development of endometrial cancer cells and induced apoptosis by upregulation of cleaved caspase‐3 and PARP and downregulation of BCLXL, XIAP, and pAKT expression.^27^

## 5 NANOFORMULATION STRATEGIES OF ESCULETIN WITH AIMING BETTER BIOAVAILABILITY

Nanotechnology has revolutionized drug delivery systems, offering numerous advantages such as increased drug solubility, improved stability, targeted delivery, and enhanced bioavailability. When it comes to cancer treatment, these advantages are incredibly crucial. Formulating anticancer agents like esculetin into nano‐sized particles can significantly improve their efficacy. The research was carried out to examine the impact of nano‐esculetin on insulinoma INS‐1 cells, with an in vitro assessment of its effect on cell death and associated mechanisms.^90^ According to the findings, esculetin can exhibit anti‐tumor activity after being loaded into PLGA nano‐micelles, and nano‐encapsulation increases its cytotoxic action in vitro with 92% encapsulation efficiency.^90^ Lu et al stated that the electrochemical response of esculetin was significantly improved by the addition of CdSe NPs to PDDA‐G over a range from 1.0 × 10^−8^ to 5.0 × 10^−5^ mol L^−1^ with a detection limit of 4.0 × 10^−9^ mol L^−1^ (*S*/*N* = 3).^91^ Administration of esculetin with zinc oxide nanoparticles elevated caspase‐3 expression.^92^ When functionalized with the coumarin molecule, esculetin, amorphous calcium phosphate nanoparticles (ACP NPs) exhibit outstanding biocompatibility, and biodegradability features.^93^ ACP NPs demonstrated a more focused cytotoxicity against colorectal cancer (CRC) cells (T‐84 cells) with an IC50 of 71.42 g/mL despite not harming human blood cells.^93^ A quick and environmentally friendly method was used to successfully create the nanocomposites of titanium dioxide nanoparticles adorned poly (diallyldimethylammonium chloride)‐functionalized grapheme (TiO2‐PDDA‐Gr) that optimize the efficiency of esculetin.^94^ The purpose of the study performed by Song et al was to examine the protective effects of esculetin against neurotoxicity brought on by intragastric administration of zinc oxide nanoparticles (ZnO NPs) in Sprague–Dawley (SD) rats, which showed positive results.^95^ According to these results, esculetin may be integrated into solid, stable nanoparticles to enhance biological processes. A summary of nano‐esculetin in cancer treatment is tabulated in Table 3.

**TABLE 3:** Summary of nano‐esculetin in cancer treatment.

Study object	Key finding	References
Nano‐esculetin on INS‐1 cells	↑ Anti‐tumor activity ↑ Cytotoxic action in vitro with 92% encapsulation efficiency	^90^
Esculetin‐CdSe NPs	↑ Efficacy of esculetin	^91^
Esculetin‐ZnO NPs	↑ Caspase‐3 expression	^92^
	↑ Efficacy of esculetin	^95^
Esculetin‐ACP NPs	↑ Cytotoxicity against colorectal cancer	^93^

## 6 POTENTIAL SYNERGY OF ESCULETIN WITH OTHER AGENTS IN CANCER TREATMENT

The devastation caused by cancer is widespread, and natural remedies have previously been shown to be highly effective in treating cancer. Combined natural treatments have shown a significant benefit against cancer and decreased the adverse effects of several medications.

Recent studies of esculetin revealed the pharmacodynamic interactions between esculetin and six commonly used cytostatic drugs (cisplatin, epirubicin, docetaxel, paclitaxel, mitoxantrone, and vemurafenib) are very effective for cancer treatment. Epirubicin, and vemurafenib showed antagonistic interactions with esculetin, while cisplatin, docetaxel, and paclitaxel showed additive interactions with esculetin. Combination of these drugs with esculetin decreases cell viability, cell proliferation, and cytotoxicity, including arrest of the cell cycle in the G1 phase, induction of cytochrome c, p53, p21, and p27 expression, reduction of CDK2 and CDK4 expression and prevention the binding interaction between Nrf2 and KEAP‐1 in the range of concentrations of 2–200 μM in human malignant melanoma cell lines (FM55P, A375, FM55M2, and SK‐MEL28).^96^ Morin, and esculetin supplementation effectively target tumor metabolism via β‐cateinin/c‐myc signaling and affect glycolysis and glutaminolysis to abrogate colon cancer in rats.^47^ Regarding treating leukemia, the combined use of esculetin, and HA14‐1 effectively induced Bid cleavage and loss of mitochondrial membrane potential (MMP, Δψm), leading to the activation of caspases and cleavage of poly(ADP‐ribose) polymerase (PARP) in Bcl‐2‐overexpressing (U937/Bcl‐2) cells and upregulated the expression of death receptor 4 (DR4), and activation of extracellular‐regulated kinase (ERK) in a time‐dependent manner. Furthermore, the apoptosis property of esculetin is mediated by HA14‐1‐induced, which can reverse Bcl‐2 anti‐apoptotic action via a mitochondrial‐mediated pathway.^61^ Again, in HCC, SMMC‐7721 cells, esculetin combined with sorafenib have a significant effect. By modulating the EGFR and VEGF‐RAS/ERK/PI3K/NF‐Baxes along with a significant upregulation of the apoptotic p38MAPK/caspase‐3 axis, modulation of pI3k/p38MAPK crosstalk, and inhibition of the proliferation marker Ki67, sorafenib, and esculetin combination exerted a potent synergistic anti‐tumor effect. This work sets the road for the potential future treatment of HCC involving esculetin as a potent adjuvant of sorafenib.^56^

A summary of the combined effect of esculetin with other phytochemical and chemotherapeutics agents is shortlisted in Table 4.

**TABLE 4:** Overview of synergistic activity of Esculetin with other phytochemicals and conventional chemotherapeutic agents.

Cancer type	Combined agents	Cell line	Combined target	References
Melanoma	Cisplatin Epirubicin Docetaxel Paclitaxel Mitoxantrone Vemurafenib	FM55P, A375, FM55M2 and SK‐MEL28	↑ p53, p21 and p27 expression ↓ CDK2 and CDK4 expression ↑ Arrest G1 phase	^96^
Colon cancer	Morin	Rat model	↓ β‐catenin	^47^
Leukemia	HA14‐1	U937 cells	↑ ERK, DR4 ↓ Bcl‐2	^61^
Hepatocellular carcinoma	Sorafenib	SMMC‐7721	↓VEGF‐RAS/ERK/PI3K/NF‐Baxes ↑ caspase‐3	^56^

## 7 POWER OF ESCULETIN IN ALLEVIATING MULTIDRUG RESISTANCE IN NUMEROUS CANCER TYPES

A significant contributor to the failure of many types of chemotherapy is multidrug resistance, the main mechanism by which many malignancies develop resistance to chemotherapy medications.^97^ Patients with solid tumors and blood malignancies, such as breast, ovarian, lung, and lower gastrointestinal tract cancers, are affected.^97^ The defense mechanism, such as drug efflux,^98^ may be the source of this resistance mechanism, besides drug inactivation,^99^ drug detoxification,^99^ drug target modification,^100^ involvement of cancer stem cell,^101^ miRNA dysregulation,^102^ epigenetic alterations,^103^ irregular DNA damage/repair mechanism, tumor microenvironment, modulating ROS,^101^, ^103^ can be the origin of this resistance. Many proteins, including P glycoprotein (P‐GP), MRP 1, MRP 1‐9, BCRP, and changes in beta‐tubulin, are linked to the occurrence of drug resistance.^6^ A literature study shows various drugs have been developed and employed to combat multidrug resistance. However, the bulk of these were ineffective for the intended use due to their numerous side effects. Esculetin alleviates drug resistance by downregulating cyclin D1 in breast cancer (CMT‐U27, CF41.mg),^38^ colon cancer (HCT‐116 cell),^45^ leukemia (HL‐60),^60^ melanoma (G361 cell),^24^ and renal cancer (786‐O and SN12‐PM6 cell).^26^ Esculetin dramatically reduced p65‐NF‐κB and ROS levels, improving anticancer drug sensitivity in resistant pancreatic cancer cell lines.^18^ Besides, it reduced p65‐NF‐κB in leukemia as well, while administered with 20 μM that improving the sensitivity of the anticancer drug.^60^ Esculetin was more effective in colon cancer,^16^ leukemia,^64^ and endometrial cancer^27^ through downregulating Bcl‐2, simultaneously increasing Bax, and mitigating PI3K/AKT signaling pathway in liver cancer via reducing drug efflux,^17^ and prostate cancer.^25^ Esculetin activates Nrf2 signaling pathway in liver cancer in vitro HepG2, and in vivo male C57BL/6J mice.^52^ Furthermore, esculetin has the potential to be used in conjunction with chemotherapy treatments against breast cancer since it induces cytotoxicity, promotes cell cycle arrest through increasing caspase‐3, and caspase‐9 while suppressing CDK1, and cyclin B1 expression with a dose of 60 μM.^15^

To create novel drug molecules against cancer that are resistant to several drugs, esculetin may be further investigated as a lead compound.^104^

## 8 ADVERSE EFFECT AND CONTROVERSY OF ESCULETIN

Even though esculetin claims to have hundreds of therapeutic benefits, it may also address inevitable negative consequences or toxic reactions. Recently, esculetin, a physiologically active coumarin, was isolated from the bark of *Fraxinus japonica* BLUME.^105^ When mice were treated with 1500 mg/kg of esculetin by the intraperitoneal route, some acute effects were shown in the median lethal dose test.^105^ While testing anti‐hepatitis B virus activity of esculetin in vitro and in vivo, the toxicity was also checked.^106^ Following 21 days of therapy, there was no discernible difference between the esculetin, and model control groups regarding feather color, body weight, food intake, irritation response, or mental state. It proves that esculetin has no overtly harmful short‐term effects on ducks.^106^ Wu et al found that the in vivo toxicity of esculetin was negligible at 50 mg/kg while treating HepG2 cells with target protein phosphoglycerate kinase 2 (PGK2), glycerol‐3‐phosphate dehydrogenase (GPD2) and glucose‐6‐phosphate isomerase (GPI).^107^ Esculetin reduces blood triglyceride levels, prevents low‐density lipoprotein (LDL) from oxidizing, and raises the level of high‐density lipoprotein cholesterol that leaves the body (HDL‐C) while treating atherosclerosis without showing any toxicity, hence proving it is a safe and reliable, easy to be absorbed by the body.^108^ Witaicenis et al summarized that esculetin has a better pharmacological profile and has only mild side effects, making it a potential alternative treatment for IBD.^109^ Administration of esculetin does not cause any allergic reaction or skin inflammation rather, when given orally, esculetin lessened the signs of atopic skin irritation brought on by DFE/DNCB by suppressing the expression of Th_1_, Th_2_, and Th_17_ cytokines.^110^ Rather, esculetin has numerous therapeutic effects against colitis, ulcerative, diarrhea,^111^ dyslipidemias,^112^ fatty liver, hepatomegaly,^50^ hyperinsulinism, hypertension,^112^ and ventricular fibrillation.^113^ Overall, few side effects and trivial toxicity of esculetin were noticed, yet esculetin can be employed for preventive purposes since it may function as a new anticancer agent. One controversy is its high dose. Enhancing its bioavailability and reducing the total amount are crucial strategies to maximize its effectiveness in cancer treatment. Some possible approaches, including formulation optimization (nanoparticle formulations and micelles and liposomes), prodrug development, combination therapy, targeted therapy, enhancing absorption, bio‐enhancers, incorporating advanced drug delivery systems, and optimizing administration routes can significantly improve the bioavailability of esculetin and reduce the total dose required for effective cancer treatment, thereby minimizing potential side effects and improving patient outcomes.

## 9 PHARMACOKINETICS OF ESCULETIN AND FUTURE PERSPECTIVE IN DRUG DEVELOPMENT

In silico approaches conducted pharmacokinetics or ADME/Tox prediction through computational tools such as Schrodinger's QuickPro modules, online accessible server admetSAR, and SwissADME were used.^114^

**FIGURE 3:**
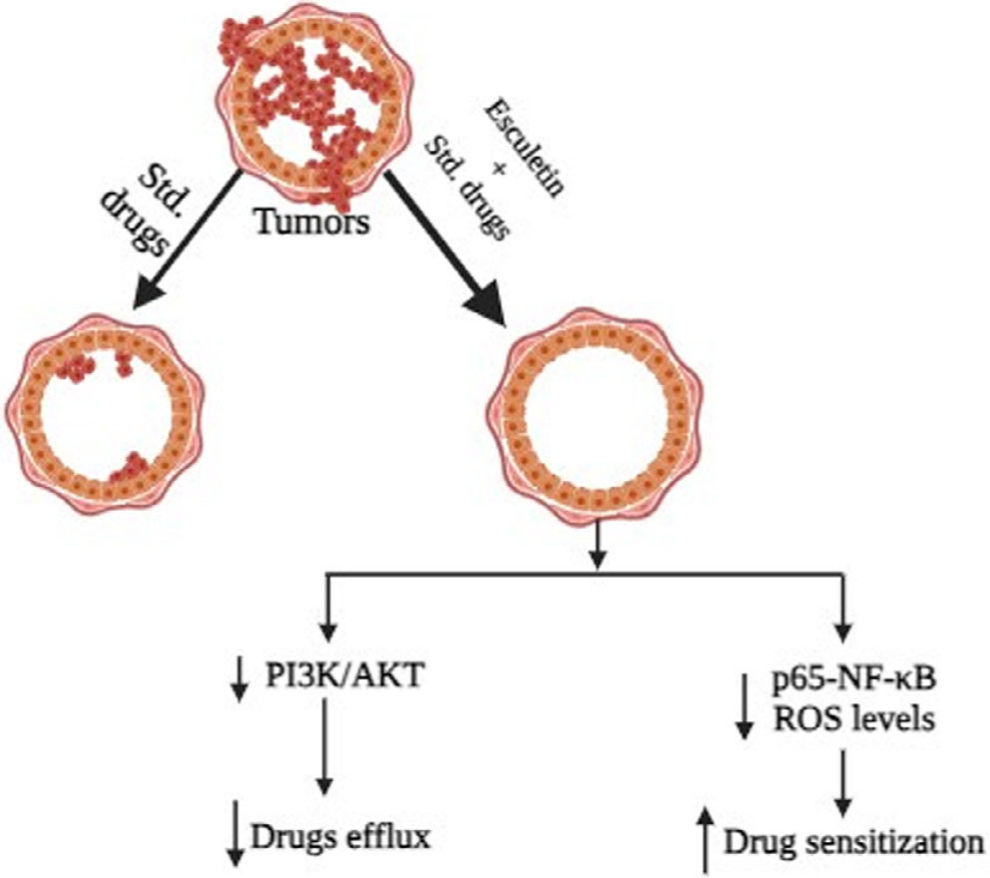
The synergistic effect of esculetin with other drugs. Using only conventional drugs recovers cancer at a minimum range, whereas using esculetin with standard drugs improves efficacies by reducing drug efflux and increasing drug sensitivity.

Using Lipinski's “Rule of Five,” the “drug‐likeness” test was conducted, and all found compounds passed the test. More precisely, it was found that esculetin's molecular weight, QPlogPo/w, HBD, and HBA were 178.030 g/mol, 0.1, 2, and 4, respectively. This indicates that the compounds have good drug‐like qualities. Esculetin's physical and chemical characteristics, such as its molecular weight (MW), volume, density, nHA, nHD, nRot, nRing, MaxRing, flexibility, stereo centers, TPSA, logS, logP, and logD, were determined using the ADMETlab 2.0 server at Figure 3.

Evaluating drug absorption, distribution, metabolism, excretion, and toxicity (ADMET) prediction may help minimize the possible danger, time, and expense of assessing whether or not a molecule is suitable to advance to the clinical stage. The pharmacokinetics profile of all identified compounds predicted using in silico tools, such as Schrodinger's QuickPro modules and the online accessible server admetSAR, which provides absorption, distribution, metabolism, excretion, and toxicity at a reliable level, is tabulated in Table 5. This profile describes the drug‐like properties of each compound. For medications to enter the bloodstream, and be used in practical tasks, their ability to absorb is crucial. According to our projected outcome, esculetin may be highly absorbed orally and pass through skin, gut, and kidney cells. Drug movement inside the body is referred to as distribution, and it depends on several variables, including the permeability of the blood–brain barrier, the permeability of the central nervous system, the drug's capacity to attach to plasma proteins, and the overall volume of distribution. Drug concentration in the bloodstream significantly decrease when they bind to plasma proteins such as human serum albumin, lipoprotein, glycoprotein, and globulins. The notion that our proposed compounds come within the suggested range implies that they can reach the target region, and function correctly through the bloodstream. The mass of bodily fluid sufficient for blood plasma to dissolve is characterized as the volume of distribution. Admittedly, esculetin is more likely to spread evenly in tissue and blood plasma. Furthermore, the central nervous system and blood–brain barrier (QPlogBB) are critical for medications that address brain disease. Unfortunately, esculetin has a poor potential to cross the brain–blood barrier and central nervous system. The CYP450 enzyme metabolizes more than 80% of medications via liver first‐pass detoxification, and blocking this enzyme enhances drug toxicity and a multitude of side effects. Esculetin is a substrate for the majority of CYP450 subunits, including CYP1A2, CYP2C9, and CYP2D6, according to the expected metabolic outcome. Esculetin's in silico toxicity profile has been predicted based on the following parameters: hepatotoxicity, cardiotoxicity, skin sensitization, kidney toxicity, eye irritation, and AMES toxicity. Esculetin has demonstrated no toxicity against any of these except for eye irritation and skin sensitization. Using the total clearance (CLtot) and T 12, we estimated the clearance profile based on the renal clearance property of the compounds. Esculetin has the potential to be easily eliminated from the body once it has served its therapeutic purpose, according to the results, which confirmed the prediction.

**TABLE 5:** In silico drug‐likeness and pharmacokinetics properties profile of Esculetin.

		Predicted remarks		Comments
Descriptors		Predicted value	Recommended rage	
Drug likeness	Molecular weight	178.030 g/mol	130.0–500	Good
	QPlogPo/w	0.1	−2.0–6.5	Soluble
	donorHB	2	0.0–6.0	Good donor
	accptHB	4	2.0–20.0	Good acceptor
Absorption	Percent human oral absorption	69.56	>80% is high	Poorly absorbed
	Skin permeability	−3.9	−8.0 to –1.0	Highly absorbed
	Caco2 permeability	221.5	25–500	Highly permeable
	MDCK permeability	1.2e‐05	NA	Permeable
	P‐glycoprotein substrate	Yes	NA	Effective
Distribution	BBB permeability	−0.921	−3.0 – 1.2	Moderate permeable
	CNS permeability	1.3	−2 to +2	Poorly permeable
	Human serum albumin	85.207%	60%	High binding affinity
	VDs	0.487	NA	Good distribution
Metabolism	CYP1A2 substrate	Yes	NA/yes	Effective
	CYP2C19 substrate	No	NA/yes	Non‐effective
	CYP2C9 substrate	Yes	NA/yes	Effective
	CYP2D6 substrate	Yes	NA/yes	Effective
	CYP3A4 substrate	Yes	NA/yes	Effective
Toxicity	Eye irritation	Yes	NA/yes	Toxic
	Hepa‐toxicity	No	NA/yes	Non‐toxic
	AMES toxicity	No	NA/yes	Non‐toxic
	hERG I inhibitors	No	NA/yes	Non‐toxic
	Kidney toxicity	No	NA/yes	Non‐toxic
	Skin sensitization	Yes	NA/yes	Toxic
Excretion	CL	16.579		
	T 1/2	0.882		

Esculetin's pharmacokinetic profile indicates strong lead and efficiency with low toxicity risks. Esculetin can, therefore, be a helpful starting point for the development of drugs for the treatment of cancer and drug discovery. Examples include docking and pharmacophore‐based virtual screening.

## 10 CONCLUDING REMARK AND FUTURE RECOMMENDATION

**FIGURE 4:**
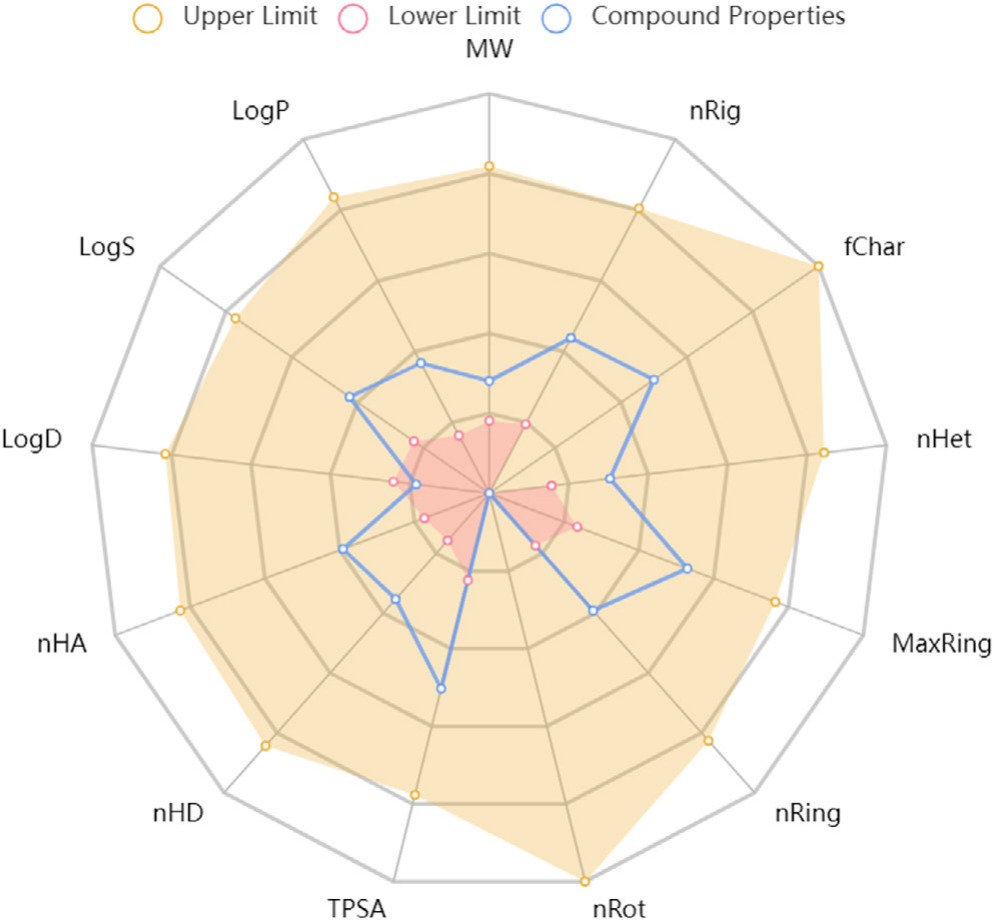
Graphical presentation of drug likeness and pharmacokinetics studies of esculetin. MW (Molecular weight), Volume (Vander Waals Volume), Density, nHA (The number of hydrogen bond acceptors), nHD (The number of hydrogen bond donors), nRot (Number of ratable bond), nRing (Number of ring), MaxRing (Number of atom in the biggest ring), nHet (Number of hetero atoms), fChar (Formal charge), nRig (number of rigid bond), flexibility, stereo centers, TPSA (Topological polar surface area), logS (Water solubility), logP (Partition coefficient), logD (Distribution co‐efficient).

Although, existing treatment strategies for cancer are failing (due to several factors), natural products are becoming influential in treating human cancer. Numerous investigations have been done to determine how esculetin works against various malignancies. In our current review, we found that esculetin inhibited the growth of many different types of cancer, including those of the breast, colon, liver, pancreatic, leukemia, lung, laryngeal, oral, salivary, melanoma, prostate, renal, endometrial, gastric, and ovaries at different doses and study models (Figure 4). Its role in cancer prevention has been proven at other pathways, such as by modulating signaling cascades involved in apoptosis, autophagy, necrosis, metastasis, angiogenesis, cell proliferation, cytotoxicity, cell growth, anti‐hepatotoxicity, nuclear shrinkage and fragmentation, oxidative stress, inflammation, and DNA damage. The capacity of esculetin to prevent cancer has been linked to the regulation of multiple proteins, including Bcl‐2, Bcl‐xL, Bax, Bak, Bid, caspases,^3^, ^7^, ^8^, ^9^, ^12^ cyclins (B1, D1, E) and CDKs,^2^, ^4^, ^6^ p21, p27, p53, and p27KIP, VEGF, GRP‐78, PERK, MMP‐2, MMP‐7, PARP, Nrf‐2, Ki‐67, pRB, N‐cadherin, E‐cadherin, survivin, sp1, JAK2/STAT3, cyto‐chrome‐C, NF‐κB, NF‐κBp65, NF‐κBp50, Wnt/β‐catenin, IL‐6, TNF‐α, phosphorylated‐Akt, PI3K/Akt, MAPK, Raf/MEK/ERK. When combined with other phytochemicals or conventional medications, esculetin may be more effective against cancer than alone. Synergistic mechanisms like this help reduce the side effects of cancer treatment medications that have been around for a while. The bioavailability of esculetin in cancer therapies may be increased by combining it with specific nanoparticles, which have been shown to be effective. In addition, this molecule has favorable pharmacokinetics while posing a lower risk of adverse effects, suggesting that it might be a suitable candidate for drug development when combined with other methods, such as network pharmacology and molecular docking, for the treatment of a variety of disorders, most notably for cancer. The authors suggest doing long‐term animal and clinical trial research to get a better understanding of the possible therapeutic advantages of treating cancer as well as the potential toxicities of the treatment. Despite the encouraging results from these studies, more research is needed to understand the anticancer potential of esculetin fully. Clinical trials are also required to confirm its efficacy in humans, and to determine the optimum dosage and treatment duration. If esculetin becomes an effective chemotherapeutics agent after passing all phases, some derivatives can be synthesized to assess their anticancer potential. Nevertheless, esculetin provides a promising lead in the quest for developing novel, and effective anticancer therapies.

## AUTHOR CONTRIBUTIONS

**Md. Rezoan Hossain:** Writing – original draft (equal). **Fatema Tuj Zahra Shova:** Writing – original draft (equal). **Munni Akter:** Writing – original draft (equal). **Shahporan Shuvo:** Writing – original draft (equal). **Nasim Ahmed:** Resources (equal). **Afroza Akter:** Writing – original draft (equal). **Munira Haque:** Writing – original draft (equal). **Md Roman Mogal:** Writing – original draft (equal). **Hasi Rani Saha:** Writing – original draft (equal). **Bidhan Chandra Sarkar:** Writing – original draft (equal). **Umme Salma:** Writing – original draft (equal). **Md Sohel:** Conceptualization (lead); supervision (lead); visualization (lead); writing – original draft (lead); writing – review and editing (lead).

## CONFLICT OF INTEREST STATEMENT

The authors have stated explicitly that there are no conflicts of interest in connection with this article.

## ETHICS STATEMENT

Not applicable.

## Data Availability

Data included in article/supplementary material/referenced in article.
